# Chromosome structure modeling tools and their evaluation in bacteria

**DOI:** 10.1093/bib/bbae044

**Published:** 2024-02-21

**Authors:** Tong Liu, Qin-Tian Qiu, Kang-Jian Hua, Bin-Guang Ma

**Affiliations:** Hubei Key Laboratory of Agricultural Bioinformatics, College of Informatics, Huazhong Agricultural University, Wuhan 430070, China; Hubei Key Laboratory of Agricultural Bioinformatics, College of Informatics, Huazhong Agricultural University, Wuhan 430070, China; Hubei Key Laboratory of Agricultural Bioinformatics, College of Informatics, Huazhong Agricultural University, Wuhan 430070, China; Hubei Key Laboratory of Agricultural Bioinformatics, College of Informatics, Huazhong Agricultural University, Wuhan 430070, China

**Keywords:** prokaryotes, chromatin interaction, Hi-C, chromosome modeling, algorithm evaluation

## Abstract

The three-dimensional (3D) structure of bacterial chromosomes is crucial for understanding chromosome function. With the growing availability of high-throughput chromosome conformation capture (3C/Hi-C) data, the 3D structure reconstruction algorithms have become powerful tools to study bacterial chromosome structure and function. It is highly desired to have a recommendation on the chromosome structure reconstruction tools to facilitate the prokaryotic 3D genomics. In this work, we review existing chromosome 3D structure reconstruction algorithms and classify them based on their underlying computational models into two categories: constraint-based modeling and thermodynamics-based modeling. We briefly compare these algorithms utilizing 3C/Hi-C datasets and fluorescence microscopy data obtained from *Escherichia coli* and *Caulobacter crescentus*, as well as simulated datasets. We discuss current challenges in the 3D reconstruction algorithms for bacterial chromosomes, primarily focusing on software usability. Finally, we briefly prospect future research directions for bacterial chromosome structure reconstruction algorithms.

## INTRODUCTION

The three-dimensional (3D) conformation of chromosomes reveals important insights into how chromosome structure and function are related, such as gene regulation [1]. The chromosome conformation capture (3C) [2] and high-throughput 3C (3C/Hi-C) [3] techniques opened a new avenue for studying the 3D conformation of chromosomes. The sequencing data obtained from 3C/Hi-C experiments can be mapped to the genome to identify the positions of both ends of the sequencing fragments. Then, the numbers of interactions between all fragments of the chromosome (comprising the interaction matrix) can be calculated. The number in the matrix represents the probability (intensity) of interaction between fragments. By analyzing the features of interaction matrix, it is found that the spatial conformation of chromosome is not random, and there is a phenomenon that certain fragments have significantly stronger interactions among themselves than with other fragments. These fragments are called topologically associating domains (TADs) [4]. TADs have been observed in different species [5–7].

**Figure 1:**
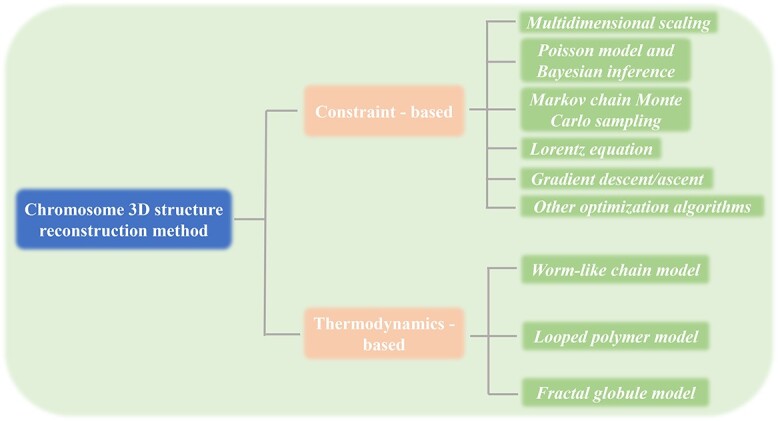
Classification of chromosome 3D structure reconstruction methods.

**Figure 2:**
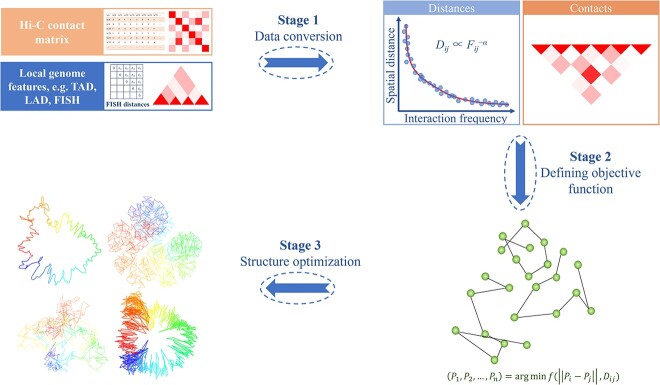
The general process of constraint-based modeling for chromosome 3D structure reconstruction. Stage 1: convert the input data (usually Hi-C contact matrix or local genomic features) into distance matrix, or directly use Hi-C contacts as constraints for modeling. Stage 2: define the objective function and constraint parameters. Stage 3: optimize the structure to obtain the final conformation.

One goal of 3D genomics is to construct the spatial structure of chromosomes by determining the spatial coordinates of each deoxyribonucleic acid (DNA) fragment or even of each nucleotide. Currently, methods for chromosome modeling are broadly classified into two categories (Figure 1): thermodynamics-based modeling and constraint-based modeling [8]. Thermodynamics-based modeling methods employ polymer physics theory and/or numerical simulation to characterize the behavior of chromatin fiber. In this approach, chromatin fibers are conceptualized as a series of polymer units, and various factors, including inter-unit interaction and conformational energy, are taken into account to determine the 3D chromosome structure [9]. This approach often uses Brownian dynamics and Monte Carlo simulation to address complex issues involving chromatin unit diffusion and movement within the cell nucleus [10]. Due to the involvement of energy optimization, this method is effective for constructing models with a small number of fragments. If the number of fragments is too large, computational speed becomes a bottleneck. Constraint-based modeling methods emphasize the utilization of experimental data or known chromosome structural information to guide the inference of chromosome structure. An increasing amount of interaction data obtained through 3C/Hi-C experiments provides constraint information on the spatial structure of chromosome. The algorithms for constraint-based modeling come in many varieties and their implementation can be roughly divided into three stages (Figure 2): transforming the interaction frequency matrix (IF matrix) into an expected spatial distance matrix or directly using Hi-C contacts as constraints for modeling, defining the objective function and constraint parameters, and optimizing the objective function to obtain the final conformation [11]. Furthermore, these two categories of modeling methods are not entirely mutually exclusive, as some algorithms appropriately combine characteristics from different methods. For instance, Yildirim *et al*. incorporated distance restraints from Hi-C interaction frequency into their plectonemic supercoiling model for *Caulobacter crescentus*, and generated an ensemble of models with base pair resolution [12].

The 3C/Hi-C technology was initially invented to investigate the 3D chromosome structure in eukaryotic organisms, yielded significant insights [3, 13, 14]. Over the past decade, it has also found extensive application in prokaryotic organisms, significantly advancing our understanding of the dynamic folding and interactions within bacterial genomes. In 2011, Umbarger *et al*. used 5C technology to produce an interaction map of *C. crescentus* with a resolution of 13 kb, and they used the early constraint-based algorithm provided by the integrated modeling platform to construct its model [15, 16]. In 2013, Le *et al*. [17] applied the Hi-C technique to obtain interaction maps and models of *C. crescentus* with a resolution of 10 kb and discovered for the first time the structural domains in bacteria similar to eukaryotic TADs, named chromosomal interaction domains (CIDs). In the same year, Cagliero *et al*. [18] obtained the first interaction map of *Escherichia coli* using 3C technology. In 2018, Lioy *et al*. [19] successfully obtained the high-quality interaction maps of *E. coli* with a resolution of 5 kb and reconstructed the spatial model using shortest-path Floyd–Warshall algorithm provided by ShRec3D software. There are also several studies on the chromosome organization of *Bacillus subtilis*. In 2015, Marbouty *et al*. [20] used the Hi-C technique to study *B. subtilis* and constructed a chromosome structure model using ShRec3D. Combined with super-resolution microscope data, they revealed the folding patterns of chromosomes during replication initiation, chromosome organization and DNA separation. Subsequently, Wang *et al*. conducted analysis on many interaction maps from *B. subtilis* cells, and uncovered the role of structural maintenance of chromosomes (SMC) complexes in chromosome compaction and replication processes [21, 22]. In a recent study, it was discovered that the Rok protein in *B. subtilis* forms anchored chromosomal loops at Mb scale, physically segregating large chromosomal regions [23]. With the emergence of an increasing number of bacterial 3C/Hi-C datasets [24–28], it is highly desired to have a recommendation on the 3D structure reconstruction tools to facilitate the prokaryotic 3D genomics. In this work, we compare all currently available tools (to the best of our knowledge) based on the same criteria.

## CURRENT CHROMOSOME STRUCTURE MODELING METHODS AND TOOLS

### Constraint-based modeling

The constraints used in chromosome modeling are mainly derived from the DNA contact maps obtained through 3C/Hi-C experiments, and the specific locus information from fluorescence microscopy. Among them, Hi-C data encompass genome-wide capture of chromosome contact probabilities, and thus have the characteristics of high throughput and large scale. However, their spatial interpretation is relatively intricate. Conversely, fluorescence locus information, such as fluorescence *in situ* hybridization (FISH) data, allows for the estimation of spatial distances between one or more pairs of distal loci, thereby explicitly indicating the physical proximity within genomic regions. Over the years, various algorithms have been proposed for inferring chromosome 3D structures from Hi-C contact data (Figures 1 and 2). Below is a brief overview.

#### Multidimensional scaling

One common chromosome modeling approach is to convert DNA interaction frequencies into their spatial distances as constraints and then iteratively optimize the locus coordinates to find a conformation that satisfy these constraints. Using a distance matrix, multidimensional scaling (MDS) analysis can be performed. MDS is a multivariate statistical technique designed to map data from a high-dimensional space to a lower-dimensional space while preserving the distance relationships between original data points as much as possible. Depending on the nature of the optimization objective, MDS can be classified into two categories: metric MDS and non-metric MDS (NMDS) [29, 30].

The objective of metric MDS is to find a configuration of points in a multidimensional space [31]. It allows inferring the coordinates of points based on the given pairwise Euclidean distances between points. In the process of inferring 3D structures of chromosomes using metric MDS, the initial step involves assigning an expected spatial distance to each pair of DNA fragments (beads). This expected distance is derived from the DNA interaction matrix. Subsequently, all beads are positioned within 3D space to minimize the objective function, which is to ensure that the Euclidean distance between each pair of beads is as close as possible to the desired spatial distance. For example, Tanizawa *et al*. [32] used this method to reconstruct the chromosome model of fission yeast and included various restrictions, such as the minimum and maximum distances between adjacent beads [33], the minimum distance between an arbitrary pair of beads [34] and specific restrictions involving centromere, telomere and the localization of ribosomal RNA coding region. However, the disadvantage of this method is that the function optimization results are dominated by pairs of beads with larger desired distances (smaller contact numbers, which are less reliable than larger contact numbers in experiments). To overcome this issue, Varoquaux *et al*. [35] proposed a variant that weights the contribution of a pair of beads (*i*, *j*) to the objective function inversely proportional to the squared distance between the corresponding beads. In addition, ChromSDE proposed an optimization method based on metric MDS that maximizes the distances between pairs of beads without any interaction frequency data, by adding a regularization term to the weighted objective function [36]. Shavit *et al*. [37] developed an R package, FisHiCal, which integrates Hi-C and FISH data to perform FISH-based iterative Hi-C calibration. Next, the calibrated Hi-C data is used in the form of distances as input for the stress minimization function. Stress minimization is a common optimization technique for metric MDS, where it infers the 3D structure by adjusting the positions of data points in a lower-dimensional space.

Non-metric MDS provides another method for chromosome structure reconstruction. This method only uses the relative ranking information of data points in the distance matrix to construct a low-dimensional representation. For example, Ben-Elazar *et al*. [38] proposed a structural prediction method based on the assumption that if the observed contact frequency between a pair of beads *i* and *j* is higher than that between a pair of beads *k* and *v*, then the pair (*i*, *j*) should be closer in 3D space than the pair (*k*, *v*). Varoquaux *et al*. [35] proposed an optimization method to solve the NMDS problem by minimizing the Shepard–Kruskal scale cost function.

#### Poisson model and Bayesian inference

Hi-C data is known to exhibit certain systematic biases, including variations in the efficiency of enzyme digestion, GC content and sequence uniqueness at the fragment ends [39]. Taking these biases into account, Hu *et al*. [40] proposed a probabilistic model in the BACH algorithm, which converts the structural inference problem into a maximum likelihood problem. This model uses a ‘beads-on-a-string’ approach commonly used in chemistry and models the spatial distances (or contact frequencies) between beads as independent random variables following a Poisson distribution to correct known systematic biases. Similarly, Varoquaux *et al*. [35] adopted a Poisson model like that in BACH’s algorithm to estimate the maximum likelihood of the consensus structure directly. Carstens *et al*. [41] introduced a Bayesian probabilistic method that leverages the posterior probability distribution over the space of chromosome conformations and model parameters. This method combines information from single-cell Hi-C contacts and FISH measurements, providing a better definition of chromosome conformation.

However, high-resolution data often suffer from the existence of excess 0 contact counts in the contact matrix due to the sparsity of remote contacts between genomic loci [11]. Since the Poisson distribution only conforms to non-zero frequency, and if the proportion of zeros in the matrix is much higher than the theoretical probability of the Poisson distribution, the Poisson model may no longer be suitable for the data. To address this issue, Park *et al*. [42] proposed a truncated Random effect EXpression (tREX) model. This model uses truncated distribution to accommodate zeros in high-resolution data and adds a random effect component for counting. Consequently, the model exhibits strong robustness across different data resolutions.

#### Markov chain Monte Carlo (MCMC) sampling

MCMC methods have found broad application in computational biology, such as RNA [43, 44] and protein [45, 46] structure prediction, phylogenetic inference [47, 48] and sequence alignment [49, 50]. MCMC is commonly used to generate robust candidate sets of structures based on noisy distance data. For example, Park *et al*. [42] used Hamiltonian MCMC when sampling the posterior value of the 3D structure coordinate set in the tREX model. Additionally, the BACH algorithm assumes that local genomic regions of interest (namely, TADs) exhibit consistent 3D chromosome structures in cell populations, and uses MCMC to infer potentially consistent 3D chromosome structure [40].

Rousseau *et al*. [51] developed a probabilistic model based on MCMC for linking Hi-C data to spatial distances, named as MCMC5C. Unlike optimization-based methods, MCMC5C models the uncertainty of spatial distance between two loci by assuming that the number of reads across two loci follows a Gaussian distribution. MCMC5C generates collections of different structures so that subclasses of structures can be discovered, and structural attributes and their distributions can be estimated to focus on statistically reasonable differences between attributes or datasets. However, estimating the Gaussian variance of each read count is challenging for MCMC5C, because a single Hi-C contact matrix does not provide sufficient information. Furthermore, the Gaussian model in MCMC5C is derived from the common 3D chromosome structure and cannot be used to measure structural changes in chromatin.

#### Lorentz equation

Hi-C data is generated from millions of cells in a single cell line, which can result in variations in genome structure and inconsistencies in chromosome contact. Consequently, it is challenging to satisfy the limitations of chromosome contact and its corresponding distance in a single structure. To address this issue, Trieu *et al*. [52] used the Lorentz function to design an objective function, which is more robust to outliers compared to the square error function. The Lorentzian function rewards the satisfaction of consistent constraints, and meanwhile ensures that the optimization process is not overly affected by inconsistent restraints. Additionally, this function is continuous and differentiable, which allows for the use of gradient-based optimization techniques effectively. Its scalability and noise-resisting feature make it a suitable approach for constructing whole 3D genome structures that involve noisy chromosome contacts.

#### Gradient descent/ascent

Gradient descent/ascent is a widely used iterative optimization algorithm in machine learning that aims to minimize/maximize the objective function or converge to its minimum/maximum value. In the 5C3D program [53], this method is used to find the best conformation of a virtual 3D DNA strand by minimizing the mismatch rate between the expected value in the distance matrix and the actual paired Euclidean distance. The MOGEN algorithm employs a contact-based optimization method that utilizes an adaptive step-size gradient ascent approach to continuously optimize the initial structure [54]. This algorithm does not require conversion of interaction frequency into distance before structure building, but rather tries to keep the distance between two contact regions below a threshold, which enables MOGEN to resist noise in the data. In the LorDG algorithm, gradient ascent optimization with an adaptive step size is used to adjust the position (namely, the *x*, *y*, *z* coordinates) of each bead to maximize a Lorentz objective function [52]. The 3DMax program employs an adaptive gradient ascent algorithm called AdaGrad, which adapts the learning rate automatically to each target parameter [55].

#### Other optimization algorithms

In addition to the methods mentioned above, there are several alternative approaches available for the reconstruction of chromosome 3D structures. Chromosome3D utilizes a distance geometry simulated annealing (DGSA) algorithm to reconstruct chromosome 3D structures from the chromosomal desired distance based on their interaction frequency [56]. It uses Hi-C distances as constraints for the simulated annealing (SA) optimization pipeline. The algorithm then optimizes the structural energy to obtain the expected 3D structure. Zhu *et al*. [57] introduced a modeling approach based on conformational energy and manifold learning. This framework interprets the spatial organization of chromosomes as the geometric structure of manifolds in 3D Euclidean space. It achieves this by converting Hi-C interaction frequencies into neighboring affinities of gene loci and then mapping them to Euclidean space to obtain the final 3D chromosome structure. Later on, Abbas *et al*. [58] systematically integrated Hi-C and FISH data and proposed the GEM-FISH algorithm. Due to the complementary nature of Hi-C and FISH data as constraints, this algorithm improved modeling result and revealed finer details of chromosomal packing.

### Thermodynamics-based modeling

**Figure 3:**
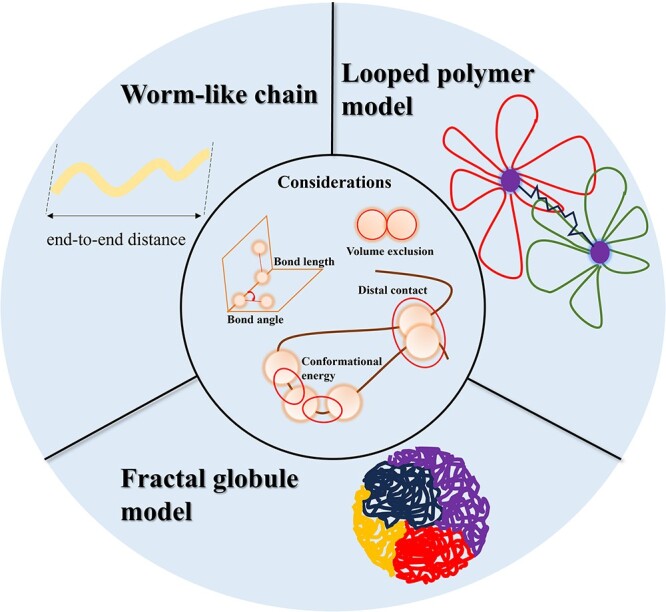
Representation of chromosomal polymer models in thermodynamic modeling. The outer layer provides an intuitive depiction of three model types, while the inner layer illustrates the factors considered during modeling.

The thermodynamics-based modeling approach considers chromosomes as polymer chains formed by the interactions of hundreds to thousands of molecular monomers. Therefore, it is essential to account for the steric configuration of molecular structures, including bond length, bond angle and volume exclusion between non-adjacent molecules. Simultaneously, the conformational energy of polymers depends on the types of interactions, which consequently influence the stability, folding states and overall structure of the polymer chain. Based on the polymer type or scale, thermodynamics-based modeling approaches can be categorized as follows (Figure 3).

#### Worm-like chain model

The worm-like chain (WLC) model is a commonly used physical model for describing the conformation of semi-flexible polymers. This model assumes that each small segment of the polymer chain can be considered as a bent ‘worm-like’ segment, connected by rigid joints. The primary concept behind the WLC model is to incorporate both the rigidity and flexibility in the chain description. Junier *et al*. [59] employed this model in combination with experimentally determined constraint parameters to perform numerical simulations of the chromosome polymer in *E. coli*. The constructed WLC model provides a coarse-grained description of protein-enveloped DNA that is used to observe chromosomal organization from a thermodynamic perspective during specific times of the cell cycle. By comparing with *in vivo* imaging data, it was revealed that the replication initiation and termination positions within structured macrodomains play a role in controlling the chromosome’s conformation and segregation.

In a research conducted by Buenemann and Lenz [60], DNA is also regarded as a semi-flexible polymer confined within the cell volume. In this context, a Monte Carlo method was developed for model testing, confirming a strong correlation between the position of gene on the chromosome and its spatial location within the cell volume. It is worth noting that while the WLC model is highly useful in capturing certain aspects of DNA mechanics, it simplifies some complexities of DNA behavior, such as the details involving base pairing and interactions with other molecules. In intricate scenarios or when specific interactions considered, more detailed models may be necessary.

#### Looped polymer model

To provide a better understanding of the compact folding of chromosomes, researchers have considered the possibility of introducing physical connections referred to as cis-interactions, which transform the linear arrangement of chromosomes into a looping structure [9]. The behavior of loops within polymer chains can be mathematically represented using various techniques, including topological parameters and statistical mechanics. The looped polymer model takes into account factors, such as the length of loops, the probability of loop formation and the impact of loops on the overall properties of the polymer. For instance, Sachs *et al*. [61] constructed a random-walk/giant-loop (RW/GL) model for human interphase chromosomes based on FISH data. In this model, chromatin fibers are conceptualized as undergoing random walks, and molecular motors moving along the chromatin fiber drive distal DNA regions together, resulting in the formation of large-scale chromatin loops. Subsequent analysis of FISH data led to the extension of the RW/GL model, giving rise to the multi-loop/subcompartment (MLS) model [62]. The MLS model posits that chromatin organization occurs at multiple hierarchical levels. It describes the genome as a series of looped structures, where contiguous loops form subcompartments. This hierarchical arrangement allows for efficient packaging of genetic material. Furthermore, the observed chromosome arms and subcompartments in the model align well with experimental results, indicating that polymer models are suitable for studying the 3D organization of the human interphase genome.

#### Fractal globule model

The fractal globule model is another theoretical framework for describing chromatin organization within the cell nucleus. This model, initially referred to as the crumpled globule, was proposed by Grosberg *et al*. [63], who argued that DNA confined within the cell nucleus needs an unknotted spatial structure to function effectively in a biological context. In contrast, the complex knotted structure of an equilibrium globule is deemed inadequate for maintaining the natural state of a functional biopolymer, as entanglements significantly reduce its reactivity to biochemical stress [64]. The fractal globule model suggests that chromatin organization does not exist in thermodynamic equilibrium. Instead, it maintains equilibrium through active processes, such as the active squeezing of chromatin loops by protein complexes. Later, with the application of Hi-C techniques, Lieberman-Aiden *et al*. [3] discovered that this fractal state indeed aligned with Hi-C data obtained from human cells. The fractal structure of the model implies that regions of the DNA chain that are close in 1D distance also tend to occupy adjacent regions in 3D space, enhancing accessibility to specific genomic regions within the cell nucleus, which is crucial for gene regulation and other cellular processes.

In prokaryotes, a nucleotide-resolution model of the *E. coli* chromosome, similar to the fractal globule, was described by Hacker *et al*. [65]. Due to the high complexity of the model structure, the degree of knotting could not be determined. However, in the *E. coli* model, the relationship function *P*(*s*) between the contact probability of two loci and their 1D distance along the genome satisfies the *s*^*−*1^ scaling law, consistent with the properties of the fractal globule model. This nucleotide-resolution model identified the four chromosome regions corresponding to macrodomains, and these regions automatically separated from each other and occupied specific positions within the nucleoid [65]. Inspired by the *C. crescentus* model proposed by the Laub lab [17], Hacker *et al*. [65] divided the chromosome into plectoneme-abundant and plectoneme-free regions (PFRs). PFRs (or the non-supercoiling regions) are identified based on RNAP binding site recognition from ChIP-chip data and considered corresponding to highly transcriptionally active regions. This nucleotide-resolution model represents a pioneering investigation of the physical properties of a bacterial chromosome at the nucleotide level, discussing the characteristics of a chromosome at both macroscopic and microscopic levels.

## TESTS AND EVALUATION

### List of evaluated software tools

The evaluated software tools are listed in Table 1. These tools mainly utilize Hi-C interaction matrices as the input data and demonstrate robust reproducibility and the ease of visualization in the generated structure models. These modeling algorithms are implemented with diverse programming languages, exemplifying certain representativeness within the field. Moreover, their open-source nature ensures accessibility to the source code. The software tools are all run under default parameters.

**Table 1:** Information on the software tools for chromosome 3D structure reconstruction

Software	Availability	Programing language	Installation dependency	Sampling algorithm	Input data	Output format	Output structures
EVR [66]	Yes	C, Python	Python; numpy; scipy	Error-vector resultant algorithm	Hi-C contact matrix	3D coordinates; pdb file format	Consensus
FLAMINGO [67]	Yes	R	R; parallel; mgcv; Matrix; prodlim; nlme	Low-rank matrix completion algorithm	Hi-C contact matrix	3D coordinates; txt file format	Consensus
GEM [57]	Yes	Matlab	MATLAB Compiler	Adaptive gradient descent method	Hi-C contact matrix; genomic loci file	3D coordinates; txt file format	Ensemble
LorDG [52]	Yes	Java	Java	Gradient ascent	Hi-C contact matrix	3D coordinates; pdb file format	Ensemble
miniMDS [68]	Yes	Python	Matplotlib; numpy; pymp-pypi; scikit-learn; scipy	MDS approximation algorithm and Kabsch algorithm	Hi–C contact matrix	3D coordinates; tsv file format	Consensus
MOGEN [54]	Yes	Java	Java	Gradient ascent	Hi-C contact matrix	3D coordinates; pdb file format	Ensemble
sBIF [69]	Yes	C++	CMake	Gradient ascent	Hi-C contact matrix	3D coordinates; txt file format	Ensemble
ShNeigh [70]	Yes	Matlab	MATLAB compiler	Shortest-path Floyd-Warshall algorithm and local proximity modeling	Hi-C contact matrix	3D coordinates; txt file format	Consensus
ShRec3D [71]	Yes	Matlab	MATLAB compiler	Shortest-path Floyd-Warshall algorithm	Hi-C contact matrix	3D coordinates; xyz file format	Consensus
SIMBA3D [72]	Yes	Python	Numpy; scipy	BFGS method with analytical gradient	Hi-C contact matrix	json file format	Consensus
Pastis [35]	Yes	Python	Python; numpy; scipy; scikit-learn; pandas	Optimization (MDS1, MDS2) and probabilistic modeling (PM1, PM2)	Hi-C contact matrix	3D coordinates; pdb file format	Consensus
TADbit [73]	Yes	Python	Matplotlib; numpy; scipy	Simulated annealing and Monte Carlo sampling	Hi-C contact matrix	3D coordinates; txt file format	Ensemble
3DMax [55]	Yes	Java, Matlab	Iava or MATLAB compiler	Gradient ascent	Hi-C contact matrix	3D coordinates; pdb file format	Ensemble

Note: Output structures (column 8): ‘Consensus’-based methods generate a single structure from the entire Hi-C dataset; ‘Ensemble’-based methods generate multiple 3D structures that satisfy the constraints from Hi-C data.

Among all the tools evaluated, EVR is the only tool specifically developed for modeling bacteria chromosome. However, some software originally designed for eukaryotes have also been used to reconstruct the 3D structure of chromosomes of various bacterial species (Table S1). The installation instructions for the tested software and a list of untested software along with the reasons for not testing are presented in the Supplementary data (Supplementary File 1: S1_Software_Installation_Instructions.pdf and Table S2). Furthermore, we have packaged the tested software and their installation environments into a Docker image which can be downloaded from the Docker Hub website (https://hub.docker.com/r/binguangma/chromosome_structure_modeling_tools).

### Datasets used in evaluation

The evaluation data consisted of three datasets: publicly available chromosomal interaction data for *E. coli* (GEO accession number: GSE107301) with a resolution of 5 kb [19], *C. crescentus* (GEO accession number: GSE45966) with a resolution of 10 kb [17] and a simulated dataset of spiral ring structure [66].

### Platform and measures

In this study, we aim to evaluate the accuracy and robustness of several chromosome 3D structure reconstruction methods. Our evaluation process is underpinned by the utilization of two distinct criteria: the Pearson correlation coefficient (PCC) and the root mean square deviation (RMSD) value. To assess accuracy, we compared the chromosome 3D structure generated by inputting the 3C/Hi-C datasets of *E. coli* [19] and *C. crescentus* [17] into each software tool with corresponding published fluorescence microscopy experimental data [74, 75]. We mapped the fluorescence labeled sites onto the reconstructed structures to determine the 3D coordinates of these sites, and then calculated the PCC between the spatial distance in structure and the experimental distance measured by fluorescence microscopy. In addition, we compared the model output to randomized fluorescence microscopy data (by shuffling the measured distances between labeled loci) to show a baseline. To assess robustness, following a previous work [66], we added different levels of noise ranging from −0.5 × *P* × IF_max_ to 0.5 × *P* × IF_max_ (*P* is noise level and IF_max_ is the maximum value in IF matrix) to the IF matrix generated from the structure of spiral ring, and then used the noisy data for 3D structure reconstruction using the software tools described above. We compared the reconstructed structure from noisy data with the original structure. At each noise level, we generated 100 sets of data for structural reconstruction and calculated the average RMSD value.

We also evaluated the running speed of the software tools, which is an important factor affecting user experience. We generated the IF matrix of spiral ring ranging from 100 to 2000 bins and used the software tools to reconstruct the 3D structure. For each bin number, we generated 10 groups of data and compared the running time on the same platform. Except for algorithms implemented in MATLAB that can use its new feature to support automatic parallelization, all other tools use CPU. The platform used was an Ubuntu18.04 (64-bit) system with Intel(R) Core (TM) i5-2400 CPU at 3.10 GHz and 20 GB DDR3 1600 MHz memory.

### Accuracy

**Figure 4:**
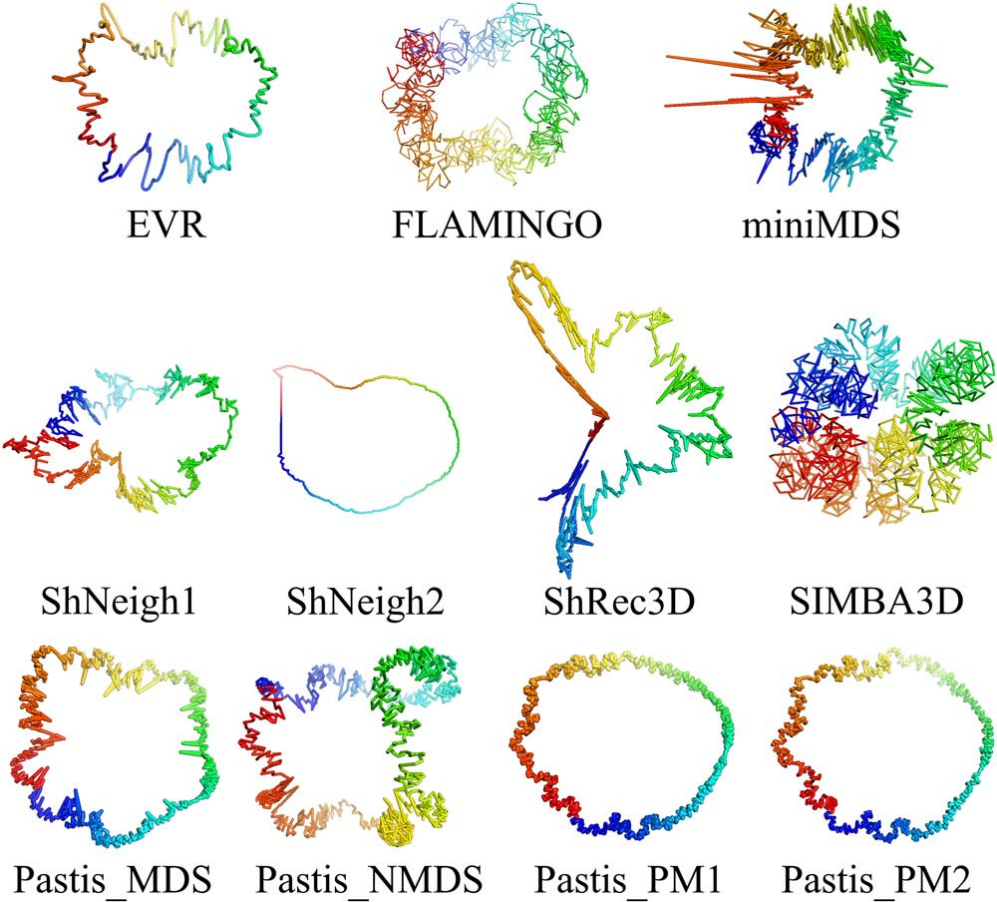
Illustration of 3D models of the *E. coli* chromosome reconstructed using eleven consensus-based algorithms.

**Figure 5:**
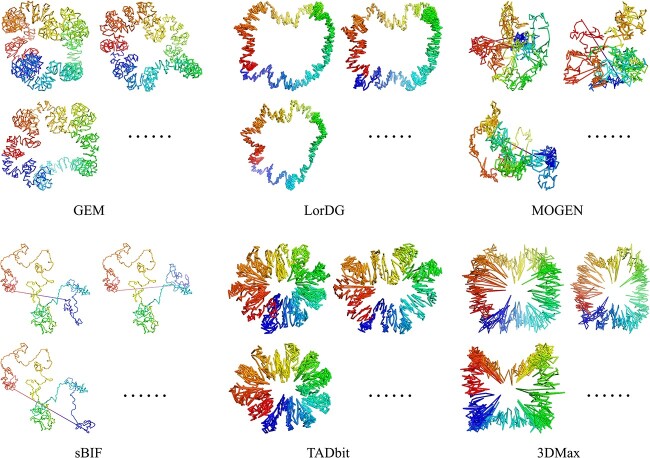
Illustration of 3D models of the *E. coli* chromosome reconstructed using six ensemble-based algorithms.

The accuracy of each modeling software was assessed by comparing the reconstructed 3D structures of the two bacterial chromosomes with corresponding fluorescence microscopy data (Table 2 and Figure S1). For the six ensemble-based reconstruction software, the distribution of PCC values for the output models was shown in Figure S2. Moreover, for a visual comparison, the 3D models of *E. coli* chromosome reconstructed using different tools are illustrated in Figures 4 and 5.

**Table 2:** Evaluation of modeling accuracy based on fluorescence microscopy data

Algorithm	*E. coli*			*C. crescentus*		
	PCC	*P*-value	Random_PCC	PCC	*P*-value	Random_PCC
EVR	0.8694	1.08E-10	0.1538	0.9148	3.59E-42	0.1121
FLAMINGO	0.7622	4.00E-07	0.1200	0.8010	3.05E-19	0.1311
GEM	0.8328	3.27E-08	0.1764	0.8866	8.68E-25	0.1097
LorDG	0.8089	2.21E-07	0.1817	0.9330	5.39E-43	0.1222
miniMDS	0.7963	5.16E-06	0.1588	0.8144	1.38E-24	0.1905
MOGEN	0.6157	2.46E-02	0.1020	0.4918	2.96E-02	0.0893
sBIF	0.6596	6.27E-05	−0.0625	0.1653	2.60E-01	0.0737
ShNeigh1	0.7764	1.77E-07	0.1549	0.6881	3.59E-07	0.1287
ShNeigh2	0.8307	4.00E-09	0.1652	0.7948	1.65E-14	0.1404
ShRec3D	0.6026	6.81E-03	0.1737	0.5387	7.48E-04	0.0665
SIMBA3D	0.7132	5.50E-04	0.1716	0.6883	1.30E-05	0.0668
Pastis_MDS	0.8280	8.38E-08	0.1619	0.6062	4.74E-06	0.1129
Pastis_NMDS	0.7834	7.07E-06	0.1356	0.6300	3.08E-07	0.1300
Pastis_PM1	0.8508	2.05E-08	0.1393	0.9179	1.57E-42	0.1106
Pastis_PM2	0.8466	3.99E-08	0.1466	0.9188	2.62E-42	0.1015
TADbit	0.8031	8.34E-05	0.1769	0.8438	3.20E-26	0.0725
3DMax	0.8253	9.11E-08	0.1545	0.8431	2.99E-26	0.1016

Note: Random_PCC values refer to the correlation between the model output and the randomized fluorescence microscopy data.

For the random scenario of each software, the results indicate that the PCC baseline is relatively low (Table 2), indicating that the modeling accuracy of each software is determined by its own algorithm. The EVR algorithm, designed specifically for prokaryotic chromosome reconstruction, exhibits the highest correlation between modeling and experimental distances in *E. coli* and the fourth highest in *C. crescentus*. GEM, LorDG, TADbit, 3DMax and two statistical methods using Poisson distribution (Pastis_PM1 and PM2) also perform well. Although PM2 can automatically adjust the formula to infer the optimal model compared with PM1, our test reveals small disparity between PM1 and PM2. Compared with the performance in *E. coli*, Pastis_MDS and Pastis_NMDS exhibits inferior correlation when applied to *C. crescentus*. This discrepancy may likely be attributed to the instability of these algorithms in processing data with different resolutions. Among the other algorithms evaluated, FLAMINGO, miniMDS, ShNeigh and SIMBA3D also showed moderate and significant correlations, despite not being specifically developed for prokaryotes. MOGEN, sBIF and ShRec3D displayed a relatively low correlation, likely due to its unsuitability for prokaryotes. Moreover, the default parameter configuration of some algorithms may be suboptimal for bacterial chromosome modeling, which may also contribute to their weaker correlations.

### Robustness

**Figure 6:**
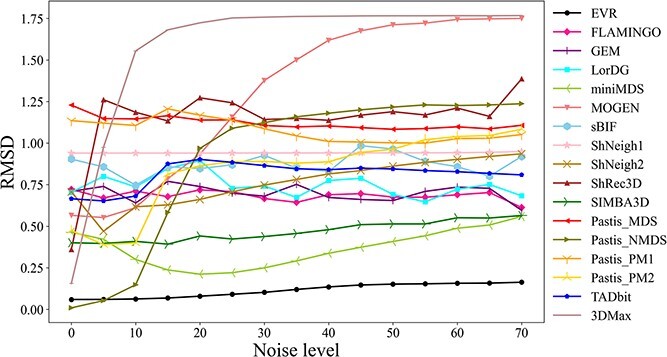
Comparison between the reconstructed structure (from noisy data) and the original structure using RMSD. Both the absolute RMSD value and its trend with the increase of noise level should be considered.

To evaluate the robustness of various chromosome 3D structure modeling tools, we generated datasets of spiral ring with varying levels of noise. As shown in Figure 6, without adding noise, the NMDS algorithm of the pastis software tool (Pastis_NMDS) performs the best, followed by the EVR algorithm, when compared with the original standard structure. Furthermore, the algorithms of 3DMax, ShRec3D, SIMBA3D, miniMDS and Pastis_PM2 all perform well, and the RMSD values of structures produced by the MOGEN, TADbit, LorDG, ShNeigh2, GEM and FLAMINGO algorithms are also at a low level. However, the sBIF, ShNeigh1, Pastis_PM1 and Pastis_MDS algorithms produce structures with relatively high RMSD values, indicating significant differences from the original structure.

After adding different levels of noise to the dataset, the EVR algorithm shows the best robustness and lowest RMSD level. The algorithms of miniMDS, SIMBA3D, FLAMINGO, GEM, LorDG, sBIF, ShNeigh, TADbit, Pastis_MDS and Pastis_ PM1 also show good robustness. However, the ShRec3D, Pastis_NMDS, Pastis_PM2, MOGEN and 3DMax algorithms demonstrate poor robustness in generating structures under different levels of noise.

### Speed

**Figure 7:**
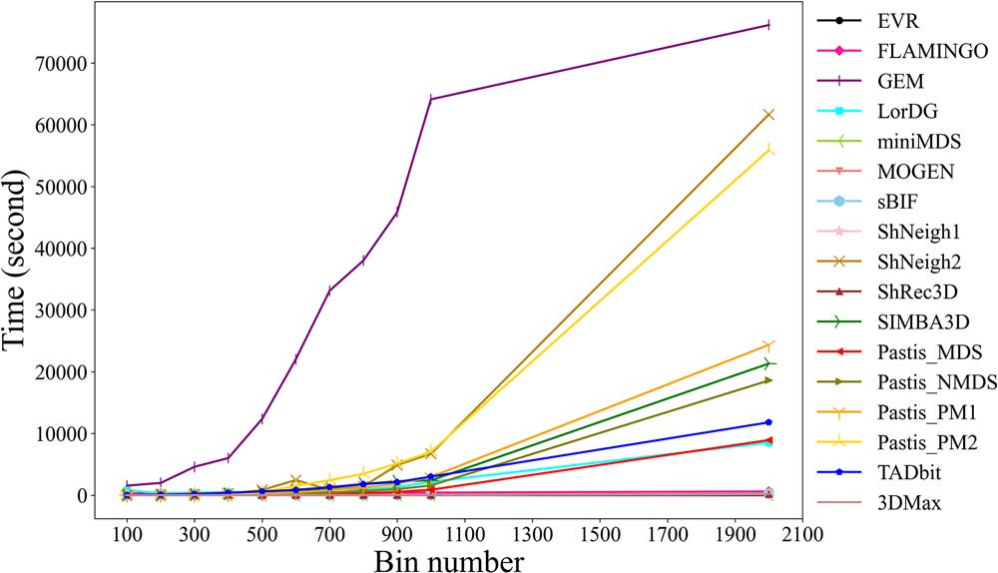
The time required to reconstruct the 3D model of the standard spiral ring structure with various bin numbers. This figure shows the time efficiency of different modeling algorithms.

We used various software/algorithms to reconstruct the 3D structure of standard spiral ring data with varying numbers of bins, and the resulting time efficiency are presented in Figure 7. The results show that the EVR algorithm is the fastest among all the tested algorithms. Additionally, the running time of EVR is not sensitive to the number of bins, within a reasonable range. The computational speed of miniMDS, MOGEN, ShNeigh1, ShRec3D, 3DMax, sBIF and FLAMINGO algorithms is slower than that of the EVR algorithm but still relatively fast. In contrast, the Pastis_MDS, LorDG, TADbit, Pastis_NMDS, SIMBA3D and Pastis_PM1 algorithms run slowly. The Pastis_PM2, ShNeigh2 and GEM algorithms are the slowest among all the tested algorithms. Moreover, the runtime of these algorithms increases significantly as the number of bins increases.

### Limitations and problems

This work provides an overview of the current state of mainstream chromosome 3D structure reconstruction software, which are predominantly written in Matlab, Python, R, Perl, Java, C++ and other languages. However, the installation and use of these software pose some challenges. First, many of the software download links are invalid or the program packages cannot be found. Second, some software requires complex dependencies that are difficult to resolve, or may encounter unknown errors when run after installation. Third, certain software may require significant parameter tuning to identify optimal parameters for specific datasets, making the exploration process time-consuming and challenging. In addition, the input and output data formats vary largely across different software tools, making it difficult to compare the modeling results.

With regards to input file format, the Hi-C interaction data are usually provided in text files with matrix or list formats. Some software requires additional data, such as restriction enzyme cutting frequency, GC content, sequence uniqueness, FISH data and Hi-C contact matrix files containing identified TADs. In prokaryotic chromosome structure modeling, some relevant data may be missing. For example, Chrom3D requires lamin-associated domain (LAD) information, making it unsuitable for modeling prokaryotic chromosomes [76].

In terms of output file format, all current modeling software generates structures containing spatial coordinate information of bins, which are usually saved in simple formats similar to PDB files, such as the modified PDB format, ‘xyz’ format, ‘mat’ format and ‘txt’ format. While some of these formats can be visualized directly through tools, such as PyMol and Chimera, others such as the txt format require additional processing and conversion before visualization. This increases the difficulty of work involved in comparing and analyzing results.

## PERSPECTIVE

### Modeling bacteria chromosome with replication forks

Bacterial chromosome replication typically originates at a single point and proceeds bidirectionally until the two replication forks converge in the terminal region [77]. Following replication initiation, active mechanisms rapidly separate the two copies [78, 79]. In eukaryotes, experiments have been conducted to distinguish between sister chromatids. As sister chromatids share the same base sequence during replication, it can be challenging to differentiate the chromosomal interactions they are involved in. To address this issue, researchers have proposed the sister-chromatid-sensitive Hi-C (scsHi-C) [80] and SisterC [81] methods. The former introduces a sister-chromatid-specific marker and culture cells in the presence of DNA nucleotide analogs. A round of DNA replication is performed to label the Watson and Crick strands in the two sister chromatids. The latter utilizes a combination of Hi-C with 5-bromo-2′-deoxyuridine (BrdU)-incorporated DNA and Hoechst/UV treatment to distinguish the interactions between sister chromatids (inter-sister interactions) and along individual sister chromatids (intra-sister interactions). Espinosa *et al*. [82] proposed a high-throughput method for monitoring sister chromatid contact (Hi-SC2). Using a multi-chromosome species *Vibrio cholerae* as a model, they monitored local variations in sister chromatid cohesion at high resolution throughout the genome. In terms of modeling, Wasim *et al*. [83] clarified the multi-scale organization of *E. coli* chromosomes at different replication stages by integrating the beads-on-a-spring model and the Hi-C interaction matrix. However, no software has been developed yet to construct a chromosome model in replication directly from Hi-C interaction data.

### Integration of structure models with multi-omics data

With the advancement of technologies, such as 3C and Hi-C, there has been increasing attention to the impact of the unique 3D structure of bacterial chromosomes on their metabolic activities. Recently, modeling algorithms have been used to reconstruct bacterial chromosome 3D structures, resulting in successful construction of more such structures [12, 17, 24, 26]. Integration of chromosome 3D structure data with other data has become a new research trend. Several studies have already combined chromosome sequencing data with other data sources. For instance, Xie *et al*. [84] integrated the genome conformation capture data of *E. coli* with its genome, biological pathway, and protein interaction data, leading to the discovery of the spatial characteristics of *E. coli* genome organization. Meanwhile, Hołówka and Płachetka [85] used molecular biology techniques along with high-throughput DNA sequencing methods to analyze bacterial chromosome structures in a precise manner at both local and global scales. Tian *et al*. [86] investigated the spatial organization characteristics of bacterial transcriptional regulatory network (TRN) using gene regulation and chromatin interaction data. Under different physiological conditions, the spatial organization features of bacterial TRNs remain relatively stable. The research results provide new insights into the connection between transcriptional regulation and chromosome spatial organization in bacteria.

### Dynamics of chromosome structure

Chromosome organization and dynamics have primarily been studied in eukaryotes due to their observable aggregation and separation by microscopy. In eukaryotic cells, 3C techniques have been used to analyze the dynamics of high-order chromosome structures, resulting in significant progress. For instance, Nagano *et al*. [87] utilized high-throughput single-cell Hi-C to generate and analyze single-cell contact diagrams of thousands of cells, revealing high genome folding heterogeneity at the single-cell level. The study confirmed that the high heterogeneity is a product of deterministic dynamics and random effects and that the cell cycle has the greatest influence on the deterministic dynamics of embryonic stem cells in mice. Recent Hi-C studies have also demonstrated that enhancer-promoter interactions and gene expression are dynamically regulated by high-order chromosome topologies [88].

Initially, due to methodological limitations, bacterial chromosomes or nucleoids were thought to be unstructured entities divided into ill-defined supercoiled domains that are randomly deposited in cells [89]. With the advent of living cell fluorescence microscopy [90], it was discovered that bacterial chromosomes are dynamic and highly organized. Current research aims to understand the molecular mechanisms behind bacterial chromosome structuring and its dynamics [91]. Le *et al*. [17] found that supercoiling is an important factor in genome compaction and helps establish CIDs *in vivo*. CIDs could potentially promote chromosome separation by preventing newly replicated chromosome entanglement during or after DNA replication. On the other hand, the loop extrusion initiated by bacterial SMC complexes is also an important mechanism affecting chromosome organization and separation [92]. The SMC complexes, loaded on a cluster of ParS sites near bacterial replication origin, extrude loops symmetrically along the entire left and right arms of the chromosome, which may help package DNA into a rod-shaped structure similar to a thin chromatid [93]. Although the loop extrusion model was proposed more than 20 years ago, there is still no suitable bacterial chromosome reconstruction (or polymer) model to explain the relevant structural dynamics. Recent advances in 3C, Hi-C, and single-cell Hi-C technology will provide new possibilities for the study of bacterial chromosome dynamics. For example, whether the reconstructed 3D model of bacterial chromosome could elucidate the driving forces (such as phase separation) and corresponding mechanisms of chromosome organization remains to be answered. Moreover, the model-based structural dynamics of bacterial chromosome in the cell cycle is still a relatively unexplored area. Future research should focus on this direction.

**Key Points**

- 3D structure reconstruction algorithms are powerful tools for studying chromosome high-order organization and its function.
- Based on the underlying computational model, 3D structure reconstruction algorithms are classified into two categories for overview.
- Currently available modeling tools are assessed on bacterial Hi-C datasets and simulated datasets to show their performance.
- Modeling the dynamic structure of bacterial chromosomes remains challenging.

## Supplementary Material

S2_Supplementary_information_bbae044

S1_Software_Installation_Instructions_bbae044

## Data Availability

The tested software and their installation environments have been packaged into a Docker image that can be obtained through the following link: https://hub.docker.com/r/binguangma/chromosome_structure_modeling_tools.

## Author Biographies

**Tong Liu** is a graduate MSc student at the Bioinformatics Center at Huazhong Agricultural University. His expertise is bacteria 3D genomics.

**Qin-Tian Qiu** is a MSc student at the College of Informatics at Huazhong Agricultural University. His research is currently focused on bacteria systems biology.

**Kang-Jian Hua** is a graduate student at the College of Informatics at Huazhong Agricultural University. His expertise is computational biology.

**Bin-Guang Ma** is a professor at the Bioinformatics Department at Huazhong Agricultural University. His expertise is systems and synthetic biology.
