# Supplementary material for: Chromosome structure modeling tools and their evaluation in bacteria
Source: Brief Bioinform. 2024 Feb 21;25(2):bbae044. doi: 10.1093/bib/bbae044 (PMC10883143; doi:10.1093/bib/bbae044)
Supplement: S2_Supplementary_information_bbae044 [file s2_supplementary_information_bbae044.pdf]

Chromosome structure modelling tools and their evaluation in bacteria

Tong Liu[#], Qin-Tian Qiu[#], Kang-Jian Hua, Bin-Guang Ma*

Hubei Key Laboratory of Agricultural Bioinformatics, College of Informatics,
Huazhong Agricultural University, Wuhan 430070, China

[#] Equal contribution.
* Corresponding author. Tel & Fax: +86 2787280877.
E-mail address: mbg@mail.hzau.edu.cn (Bin-Guang Ma)

Supplementary information

Table S1. Some software originally designed for eukaryotes but have been used to reconstruct the 3D structure of chromosomes of various bacterial species

Prokaryote	Year	Number of replicons	Genome size	Resolution of interaction matrix	Tools for reconstructing chromosome 3D structure
Bacillus subtilis [1]	2015	A circular chromosome	4.2 Mb	4 kb	ShRec3D
Mycoplasma pneumoniae [2]	2017	A circular chromosome	0.8 Mb	10 kb	TADbit
Escherichia coli [3]	2018	A circular chromosome	4.6 Mb	5 kb	ShRec3D
Sulfolobus acidocaldarius [4]	2019	A circular chromosome	2.2 Mb	15 kb	ShRec3D
Sulfolobus islandicus [4]	2019	A circular chromosome	2.5 Mb	30 kb	ShRec3D
Streptomyces ambofaciens [5]	2021	A linear chromosome and a circular plasmid	8.4 Mb	10 kb	ShRec3D
Brucella melitensis [6]	2023	Two circular chromosomes	3.3 Mb	1 kb	Pastis_MDS
Streptomyces coelicolor [7]	2023	A linear chromosome, a linear plasmid and a circular plasmid	9.1 Mb	5 kb	Pastis_MDS

Table S2. List of untested software

Software	Language	Sampling algorithm	Test result	Reason for not testing
AutoChrom3D [8]	Perl	Non-linear constrained optimization	No	Not easy to install
BACH / BACH-MIX [9]	R	Gibbs sampler with hybrid Monte Carlo, and adaptive rejection sampling (ARS)	No	The program cannot be downloaded
Chromosome3D [10]	Perl	Distance geometry simulated annealing	No	The program cannot be downloaded
ChromSDE [11]	Matlab	Linear and Quadratic Semi-definite programming (SDP)	No	The program cannot be downloaded
Chrom3D [12]	Perl	Monte Carlo optimization using the Metropolis-Hastings algorithm with simulated annealing	No	Missing input data information
DPDChrom [13]	Fortran, Python	Dissipative particle dynamics	No	The program runs incorrectly
FisHiCal [14]	R	Scaling by Majorizing a Complicated Function (SMACOF) algorithm	No	Missing input data information
GEM-FISH [15]	Matlab	Gradient descent	No	FISH data missing
Gen3D [16]	C++	Adaptation, simulated annealing and genetic algorithm	No	Missing input file information
Guarnera et al. [17]	Unknown	Markov state modeling and stochastic embedding procedure	No	Unpublished code
HAS [18]	R	Generalized linear model with Hamiltonian dynamics with simulated annealing	No	The program cannot be downloaded
Hierarchical3DGenome [19]	Java	Gradient ascent and hierarchical modeling	No	Missing input file information
InfMod3DGen [20]	Matlab	Gradient ascent	No	The program runs incorrectly
ISDHiC [21]	C, Python	Markov Chain Monte Carlo sampling using Hamiltonian Monte Carlo	No	The program has an unknown error
Kalhor et al. [22]	Unknown	Conjugate gradients and molecular dynamics with simulated annealing	No	Unpublished code
MCMC5C [23]	Java	Markov chain Monte Carlo sampling using the Metropolis-Hastings algorithm	No	The program cannot be downloaded
MBO [24]	Matlab	Manopt-manifold optimization	No	Single cell data required
ShRec3D+ [25]		Shortest-path algorithm and multi-dimensional scaling (MDS) algorithm	No	Unpublished code
Tjong et al. [26]	Unknown	Simulated annealing dynamics and conjugate gradient optimization	No	Unpublished code
tRex [27]	R	Metropolis-Hastings algorithm / Gibbs sampler and Hamiltonian Markov Chain Monte Carlo	No	The program cannot be downloaded
5C3D [28]	Unknown	Gradient descent	No	The program cannot be downloaded

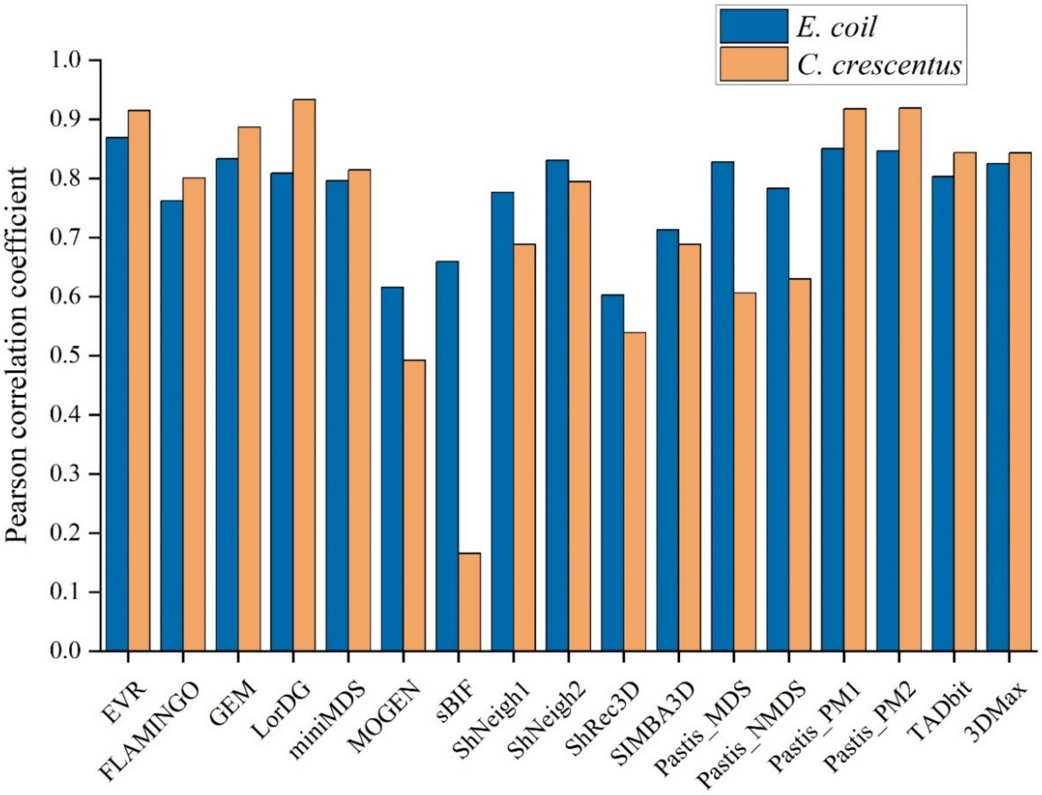

Figure S1. Barplot for the PCC of each modeling software on two bacterial datasets (see also **Table 2**).

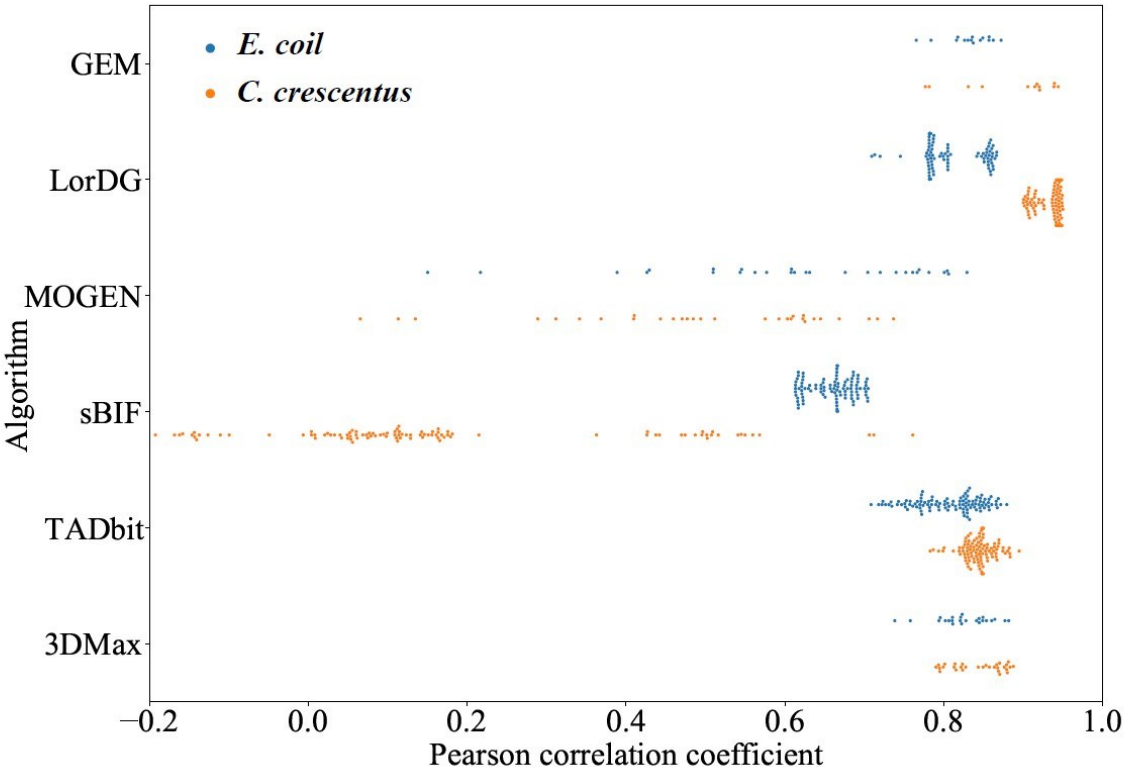

Figure S2. The distribution of PCC values for the models output by 6 ensemble-based reconstruction software tools.

References

[1] Marbouty M, Le Gall A, Cattoni DI, et al. Condensin- and replication-mediated bacterial chromosome folding and origin condensation revealed by Hi-C and super-resolution imaging. Mol. Cell 2015;**59**(4):588-602.

[2] Trussart M, Yus E, Martinez S, et al. Defined chromosome structure in the genome-reduced bacterium *Mycoplasma pneumoniae*. Nat. Commun. 2017;**8**:14665.

[3] Lioy VS, Cournac A, Marbouty M, et al. Multiscale structuring of the *E. coli* chromosome by nucleoid-associated and condensin proteins. Cell 2018;**172**(4):771-83.

[4] Takemata N, Samson RY, Bell SD. Physical and functional compartmentalization of archaeal chromosomes. Cell 2019;**179**(1):165-79.

[5] Lioy VS, Lorenzi JN, Najah S, et al. Dynamics of the compartmentalized *Streptomyces* chromosome during metabolic differentiation. Nat. Commun. 2021;**12**(1):5221.

[6] Huang YF, Liu L, Wang F, et al. High-resolution 3D genome map of *Brucella* chromosomes in exponential and stationary phases. Microbiol. Spectr. 2023;**11**(2):e0429022.

[7] Deng L, Zhao Z, Liu L, et al. Dissection of 3D chromosome organization in *Streptomyces coelicolor* A3(2) leads to biosynthetic gene cluster overexpression. Proc. Natl. Acad. Sci. U.S.A. 2023;**120**(11):e2222045120.

[8] Peng C, Fu LY, Dong PF, et al. The sequencing bias relaxed characteristics of Hi-C derived data and implications for chromatin 3D modeling. Nucleic Acids Res. 2013;**41**(19):e183.

[9] Hu M, Deng K, Qin Z, et al. Bayesian inference of spatial organizations of chromosomes. PLoS Comp. Biol. 2013;**9**(1):e1002893.

[10] Adhikari B, Trieu T, Cheng JL. Chromosome3D: reconstructing three-dimensional chromosomal structures from Hi-C interaction frequency data using distance geometry simulated annealing. BMC Genom. 2016;**17**(1):886.

[11] Zhang Z, Li G, Toh K-C, et al. Inference of spatial organizations of chromosomes using semi-definite embedding approach and Hi-C data. In: Research in Computational Molecular Biology. Berlin, Heidelberg, 2013, p. 317-32. Springer Berlin Heidelberg.

[12] Paulsen J, Sekelja M, Oldenburg AR, et al. Chrom3D: three-dimensional genome modeling from Hi-C and nuclear lamin-genome contacts. Genome Biol. 2017;**18**(1):21.

[13] Kos PI, Galitsyna AA, Ulianov SV, et al. Perspectives for the reconstruction of 3D chromatin conformation using single cell Hi-C data. PLoS Comp. Biol. 2021;**17**(11):e1009546.

[14] Shavit Y, Hamey FK, Lio P. FisHiCal: an R package for iterative FISH-based calibration of Hi-C data. Bioinformatics 2014;**30**(21):3120-2.

[15] Abbas A, He X, Niu J, et al. Integrating Hi-C and FISH data for modeling of the 3D organization of chromosomes. Nat. Commun. 2019;**10**(1):2049.

[16] Nowotny J, Ahmed S, Xu LF, et al. Iterative reconstruction of three-dimensional models of human chromosomes from chromosomal contact data. BMC Bioinform. 2015;**16**:338.

[17] Guarnera E, Tan ZW, Berezovsky IN. Three-dimensional chromatin ensemble reconstruction via stochastic embedding. Structure 2021;**29**(6):622-34.e3.

[18] Zou CC, Zhang YP, Ouyang ZQ. HSA: integrating multi-track Hi-C data for genome-scale reconstruction of 3D chromatin structure. Genome Biol. 2016;**17**:40.

[19] Trieu T, Oluwadare O, Cheng JL. Hierarchical reconstruction of high-resolution 3D models of large chromosomes. Sci. Rep. 2019;**9**(1):4971.

[20] Wang SY, Xu JB, Zeng JY. Inferential modeling of 3D chromatin structure. Nucleic Acids Res. 2015;**43**(8):e54.
[21] Carstens S, Nilges M, Habeck M. Inferential Structure Determination of Chromosomes from Single-Cell Hi-C Data. PLoS Comp. Biol. 2016;**12**(12):e1005292.
[22] Kalhor R, Tjong H, Jayathilaka N, et al. Genome architectures revealed by tethered chromosome conformation capture and population-based modeling. Nat. Biotechnol. 2012;**30**(1):90-8.
[23] Rousseau M, Fraser J, Ferraiuolo MA, et al. Three-dimensional modeling of chromatin structure from interaction frequency data using Markov chain Monte Carlo sampling. BMC Bioinform. 2011;**12**:414.
[24] Paulsen J, Gramstad O, Collas P. Manifold based optimization for single-cell 3D genome reconstruction. PLoS Comp. Biol. 2015;**11**(8):e1004396.
[25] Li JG, Zhang W, Li XD. 3D genome reconstruction with ShRec3D+ and Hi-C data. IEEE/ACM Trans Comput Biol Bioinform 2018;**15**(2):460-8.
[26] Tjong H, Li WY, Kalhor R, et al. Population-based 3D genome structure analysis reveals driving forces in spatial genome organization. Proc. Natl. Acad. Sci. U.S.A. 2016;**113**(12):E1663-E72.
[27] Park J, Lin SL. Impact of data resolution on three-dimensional structure inference methods. BMC Bioinform. 2016;**17**:70.
[28] Fraser J, Rousseau M, Shenker S, et al. Chromatin conformation signatures of cellular differentiation. Genome Biol. 2009;**10**(4):R37.
